# Long-term statin use in patients with lung cancer and dyslipidemia reduces the risk of death

**DOI:** 10.18632/oncotarget.9906

**Published:** 2016-06-07

**Authors:** Wen-Yen Huang, Chia-Hsiang Li, Cheng-Li Lin, Ji-An Liang

**Affiliations:** ^1^ Department of Radiation Oncology, Tri-Service General Hospital, National Defense Medical Center, Taipei, Taiwan; ^2^ Institute of Clinical Medicine, National Yang-Ming University, Taipei, Taiwan; ^3^ Division of Pulmonary and Critical Care Medicine, Department of Internal Medicine, China Medical University Hospital, Taichung, Taiwan; ^4^ Management Office for Health Data, China Medical University Hospital, Taichung, Taiwan; ^5^ College of Medicine, China Medical University, Taichung, Taiwan; ^6^ Graduate Institute of Clinical Medical Science, College of Medicine, China Medical University, Taichung, Taiwan; ^7^ Department of Radiation Oncology, China Medical University Hospital, Taichung, Taiwan

**Keywords:** lung cancer, statin, population-based case-control study

## Abstract

**Background:**

Clinical studies have obtained inconsistent results of statin use on cancer outcomes. This study investigated the association between statin use and lung cancer mortality.

**Results:**

The use of statin decreased mortality (hazard ratio = 0.91; 95% confidence interval: 0.86–0.96; *P* < .01). The patients with a high cumulative defined daily dose of statin use before lung cancer diagnosis exhibited a low risk of mortality.

**Materials and Methods:**

We conducted a population-based case-control study of patients with dyslipidemia. Among them, 6270 had used statins for at least 3 months before lung cancer diagnosis, and 6270 had never used statins.

**Conclusions:**

We found that statin use can reduce lung cancer mortality. A further prospective study is necessary to confirm these findings.

## INTRODUCTION

Lung cancer is the leading cause of cancer deaths worldwide and accounts for 13% (1.6 million) of the total cases and 18% (1.4 million) of the deaths [1, 2]. Variations in incidence rates and trends, genetic mutation, and prognosis are observed among countries. Generally, lung cancer incidence is higher in Western countries, but it is becoming increasingly common in Asian countries, probably reflecting differences in the degree of the tobacco epidemic and genetic background. The prevalence of EGFR mutation in adenocarcinoma patients is 10% in Western countries and up to 50% in Asian countries [3].

Statins are 3-hydroxy-3-methylglutaryl coenzyme A reductase inhibitors and are extensively used in the clinical treatment of hypercholesterolemia. Their effectiveness in the secondary prevention of coronary heart disease and stroke was documented [4]. In addition to their HMG-CoA-dependent effects, experimental evidence suggests that statins provide an oncoprotective effect *in vitro* and *in vivo* [5–7]. The possible mechanisms of statins' protective effects against cancer may be their HMG-CoA-independent effects (eg, functioning as broad-spectrum agents in disease pathways for inhibiting inflammation, immunomodulation, and angiogenesis) [8].

Some epidemiological and clinical studies have investigated the role of statins in protecting against lung cancer and promoting patient survival, yielding inconsistent results [9–11].

Previous studies have largely focused on the Western population. Extensive clinical evidence in the Asian population is lacking. Thus, we conducted a nationwide population-based case-control study to determine the effect of statin use in lung cancer patients in Taiwan. We also assessed the relationship between statin use and lung cancer mortality before and after lung cancer diagnosis.

## RESULTS

### Statin use reduces mortality

The median follow-up time was 5.20 years (range = .04–13.96). A total of 12 540 patients with hyperlipidemia and lung cancer diagnosis were recruited in this study. Among them, 6270 used statins regularly for more than 3 months before lung cancer diagnosis, whereas 6270 had never used statins. The statin and nonstatin cohorts were matched according to propensity scores. Table 1 displays the baseline demographic and clinical characteristics of the patients in the 2 cohorts. The CCI, a method of predicting the outcome and risk of death associated with numerous comorbid diseases according to their potential influence on mortality, is a valid prognostic indicator of mortality [12]. For various CCI scores or comorbidities (COPD, CAD, and stroke), no difference was observed between the cohorts. Table 1 indicates that both cohorts exhibited similar percentages for the diverse cancer treatment modalities (*P* = .4). Although the patients in the statin cohort registered a higher frequency of medical visits than did those in the nonstatin cohort, as predicted, the patients in both cohorts, who were matched according to the propensity scores, exhibited similar basic characteristics.

**Table 1:** Demographic characteristics of the study participants who used different medicines in the propensity-score-matched sample

Variables	Statin use				
	No *N* = 6270		Yes *N* = 6270		
	*n*	%	*n*	%	*p*-value
Age, years					
Median (Range)^†^	67.7	(21.8–99.5)	67.4	(28.3–92.9)	0.16
Sex					
Female	2548	(40.6)	2589	(41.3)	0.46
Male	3722	(59.4)	3681	(58.7)	
Frequency of medical visits/per year (5-years pre-lung cancer diagnosis) Median (Range)^†^	28.7	(0.35-1081)	32.0	(0.09–1113)	0.001
CCI score*					0.79
0	4686	(74.7)	4714	(75.2)	
1	985	(15.7)	964	(15.4)	
2	317	(5.06)	299	(4.77)	
3 or more	282	(4.50)	293	(4.67)	
Comorbidity					
COPD	3212	(51.2)	3201	(51.1)	0.84
CAD	2240	(35.7)	2276	(36.3)	0.50
Stroke	459	(7.32)	457	(7.29)	0.95
Treatment					0.40
Surgery alone	667	(10.6)	726	(11.6)	
Surgery+ adjuvant therapy^§^	258	(4.11)	245	(3.91)	
RT+/− systemic therapy	963	(15.4)	994	(15.9)	
Systemic therapy alone	1244	(19.8)	1214	(19.4)	
Untreated/palliative care	3138	(50.1)	3091	(49.3)	

Chi-square test, ^†^Mann–Whitney *U* test.

* CCI score = Charlson comorbidity index score;

§ adjuvant therapy, including systemic therapy, RT, and systemic therapy + RT.

Table 2 provides the results of the major mortality analysis of both cohorts. The median follow-up time in the nonstatin and statin cohorts was 5.34 (range = .19–14.0) and 5.02 years (range = .04–14.0), respectively. Person-years (PY), a measurement that entails assessing both the number of people and the amount of time for which each person participates in a study, is typically used for analyzing survival rates. During a follow-up period of 34 298 and 35 768 PY, the overall mortality rate obtained by dividing the number of mortality events by the PY was significantly higher in the nonstatin cohort than in the statin cohort (12.7 vs 11.9 per 100 PY). As expected, the age-specific incidence of mortality increased with age in both cohorts. Among all age groups, only the patients in the statin cohort aged 70–79 years demonstrated a significantly lower risk of mortality compared with those in the nonstatin cohort. The female patients in the statin cohort had a significantly lower mortality rate than that of those in the nonstatin cohort. In both cohorts, the mortality rate was higher in the patients with comorbidities than in those without comorbidities. Among the patients without CAD or stroke, those who used statins had a lower risk of mortality compared with those who did not (HR = .85, 95% CI = .78–.93 for CAD; HR = .92, 95% CI = .87–.98 for stroke). The patients receiving RT +/− systemic therapy or untreated/palliative care exhibited a significantly lower risk of mortality in the statin cohort than in the nonstatin cohort.

**Table 2:** Comparison of the incidence and HR of mortality stratified by sex, age, CCI score, and treatment according to medication status among the lung cancer patients

	Statin use						
	No			Yes			
Variables	Event	PY	Rate^†^	Event	PY	Rate^†^	HR^#^ (95% CI)
All	4340	34298	12.7	4254	35768	11.9	0.91 (0.86, 0.96)**
Age, years							
≤ 59	929	11142	8.34	842	10177	8.27	0.83 (0.65, 1.06)
60–69	1294	11175	11.6	1491	13404	11.1	0.89 (0.75, 1.06)
70–79	1594	9807	16.3	1582	10625	14.9	0.76 (0.64, 0.89)**
≥ 80	523	2176	24.0	339	1561	21.7	0.64 (0.33, 1.24)
Sex							
Female	1567	14680	10.7	1567	15553	10.1	0.85 (0.74, 0.99)*
Male	2773	19619	14.1	2687	20214	13.3	0.96 (0.87, 1.05)
CCI score¶							
0	3092	27122	11.4	3060	28196	10.9	0.95 (0.88, 1.03)
1	753	4676	16.1	718	4953	14.5	0.87 (0.62, 1.21)
2	258	1409	18.3	236	1335	17.7	0.71 (0.23, 2.25)
3 or more	239	1090	21.9	241	1284	18.7	0.86 (0.29, 2.55)
Comorbidity							
COPD							
No	2046	17529	11.7	2043	18368	11.1	1.02 (0.90, 1.14)
Yes	2294	16770	13.7	2211	17400	12.7	0.86 (0.77, 0.96)**
CAD							
No	2721	23086	11.8	2607	23818	11.0	0.85 (0.78, 0.93)***
Yes	1620	11213	14.4	1647	11951	13.8	0.83 (0.72, 0.97)*
Stroke							
No	3960	32425	12.2	3892	33828	11.5	0.92 (0.87, 0.98)**
Yes	380	1874	20.3	362	1940	18.7	0.82 (0.44, 1.53)
Treatment							
Surgery alone	141	4490	3.14	127	5022	2.53	0.54 (0.22, 1.35)
Surgery+ adjuvant therapy^§^	160	1659	9.65	150	1542	9.73	0.22 (0.05, 1.03)
RT+/− systemic therapy	762	5337	14.3	769	5835	13.2	0.53 (0.35, 0.79)**
Systemic therapy alone	807	6934	11.6	783	7019	11.2	0.78 (0.58, 1.06)
Untreated/palliative care	2470	15879	15.6	2425	16351	14.8	0.84 (0.75, 0.94)**

PY, person-years. Rate^†^, incidence rate per 100 PY. ¶: CCI score = Charlson comorbidity index score. ^§^adjuvant therapy, including systemic therapy, RT, and systemic therapy + RT. HR^#^, relative hazard ratio. *P < .05, **P < .01, ***P < .001.

### High dose of statin use reduces the risk of mortality

To investigate the relationship between long-term statin use and mortality risk, we measured the cumulative use and dosage of statin according to the cDDD. Table 3 displays the relationship between cDDD before lung cancer diagnosis and the risk of mortality. The HR value of the nonstatin cohort was set as the reference. We observed that multivariable-adjusted HRs significantly decreased in the patients with a high cDDD (cutoff value in the third quartile) compared with those of the patients with a low cDDD in 6 types of statin.

**Table 3:** HR and 95% CIs of mortality associated with cDDD of individual statins

	Event/N	PY	Rate^#^	HR*	(95% CI)
Non-use of statins	4340/6270	34299	12.7	1	(Reference)
Simvastatin					
< 290 DDD	653/955	4902	13.3	1.05	(0.91, 1.22)
≥ 290 DDD	200/322	2234	8.95	0.62	(0.48, 0.81)***
Fluvastatin					
< 620 DDD	377/489	2500	15.1	1.18	(1.07, 1.32)**
≥ 620 DDD	95/159	1121	8.48	0.64	(0.52, 0.78)***
Lovastatin					
<230 DDD	736/948	5135	14.3	1.07	(0.99, 1.15)
≥ 230 DDD	224/312	2280	9.82	0.70	(0.61,0.80)***
Atorvastatin					
< 80 DDD	1028/1496	7534	13.7	1.10	(1.02, 1.17)**
≥ 80 DDD	263/492	3754	7.01	0.53	(0.47, 0.60)***
Pravastatin					
< 300 DDD	297/389	2105	14.1	1.05	(0.94, 1.18)
≥ 300 DDD	71/128	918	7.73	0.58	(0.46, 0.73)***
Rosuvastatin					
< 590 DDD	251/434	2223	11.3	0.86	(0.76, 0.97)*
≥ 590 DDD	59/146	1060	5.57	0.42	(0.32, 0.54)***

DDD, defined daily dose; PY, person-years; Rate^#^, incidence rate per 100 PY; HR*, relative hazard ratio. The cDDD is partitioned into 2 segments by the third quartile. *P < .05, **P < .01, ***P < .001.

To investigate whether statin use benefits patients even after lung cancer diagnosis, we divided the statin use patients into low and high cDDD groups by setting the cutoff value at the median amount of statin use before lung cancer diagnosis (prediagnostic use) and after lung cancer diagnosis (postdiagnostic use). As shown in Table 4, the patients with a high cDDD of statin use before lung cancer diagnosis exhibited a significantly lower risk of mortality after adjustment, regardless of the statin dosage after lung cancer diagnosis. In addition, regardless of the statin dosage before lung cancer diagnosis, the patients with high cDDD after lung cancer diagnosis had a reduced risk of mortality (adjusted HR = .3). This suggests that continual uptake of statin even after cancer diagnosis likely benefits patients with lung cancer. These results supported the finding that long-term statin use in patients with lung cancer and dyslipidemia reduces the risk of mortality.

**Table 4:** Cox proportional hazards regression analysis for the risk of mortality in lung cancer patients that was performed by separating the patients into 4 groups according to their statin use status

Prediagnostic use	Postdiagnostic use	Total (*N* = 6270)	Event *n*	Adjusted HR^†^ (95% CI)
Low	Low	2713	2004	1 (Reference)
Low	High	436	168	0.30 (0.26, 0.36)***
High	Low	2493	1821	0.74 (0.69, 0.79)***
High	High	628	261	0.30 (0.26, 0.34)***
	P for trend			< 0.001

The median cDDD in the prediagnostic use patients was 180; the median cDDD in the postdiagnostic use patients was 80. Statin use lower than the median cDDD is considered low dose use; statin use higher than the median cDDD is considered high dose use. Adjusted HR^†^: adjusted for age, sex, and treatment. ***P < .001.

## DISCUSSION

Our results indicate that statin use in the patients with lung cancer and dyslipidemia was associated with reduced all-cause mortality and that cumulative dosages exhibited protective effects regardless of the types of statin. Long-term use of statins before lung cancer diagnosis was correlated with decreased lung cancer mortality. Notably, the patients who were treated through RT +/− systemic therapy or untreated/palliative care in the statin cohort demonstrated significantly reduced mortality rates compared with those in the nonstatin cohort. This may imply that statins exert protective effects against tumors in advanced stage patients.

The mechanism underlying the reduction of cancer mortality through statin use is unknown. Several biological studies have reported speculated mechanisms. Statins appear to inhibit the cell cycle [13], influence the mevalonate synthesis pathway, and reduce its metabolite, thereby causing tumor cell apoptosis [14, 15]. They also inhibit tumor metastasis [16, 17]. Statins may affect the functioning of p53-mutated cancer cells, generating cell apoptosis in breast cancer cells [18].

Previous studies on the relationship between statin use and lung cancer incidence have reported conflicting results. A previous study reported that women who used statins exhibited a higher risk of lung cancer [19]. Furthermore, no significant association between lung cancer and statin use has been determined [20–23]. However, several studies have reported that long-term statin use can reduce lung cancer incidence [10, 24, 25]. Nielsen et al. [26] used the Danish Civil Registration System to identify 18721 cancer patients who regularly used statins and 277204 cancer patients who never used statins. They indicated that statin use reduced cancer-related mortality (adjust HR, 0.85; 95% CI, 0.82–0.87; *P* < .001) and all-cause mortality (adjust HR, 0.85; 95% CI, 0. 83–0.87; *P* < .001). This could be observed for 13 cancer types, including lung cancer (adjust HR, 0.87; 95% CI, 0. 83–0.92; *P* < .001). Cardwell et al. [27] conducted a population-based cohort study in England and reported statin use before lung cancer diagnosis could reduce lung cancer-specific mortality (adjust HR, 0.88; 95% CI, 0.83–0.93; *P* < .001). Statin use after diagnosis could marginally statistically decrease cancer-specific mortality (adjust HR, 0.89; 95% CI, 0.78–1.02; *P* = .09). In the current study, we determined that long-term statin use before lung cancer diagnosis was associated with major protective effects against lung cancer mortality.

Side effects of statin use are relatively rare with most other classes of lipid lower agents. The most common side effects are statin-related myopathy. Myopathy occurs in approximate 10% and most are mild. Other possible side effects include hepatic dysfunction, renal dysfunction, cognitive impairment, diabetes mellitus, and so on. Approximately, 10% of patients had statins discontinued at least temporarily due to intolerance. However, most of them can tolerate statin long term after rechallenging [28]. In this study, we cannot report the distribution of side effects after long-term stain use because it is not included in the NHIRD.

The main strength of our study was the relatively large sample size of the population that was derived from a nationwide database, which has wide coverage, facilitating the tracing of medical service histories and comprehensive follow-ups. Thus, this provided adequate statistical power to examine the associations between statin use and lung cancer mortality. In addition, it was highly suitable for comparing current medication and cumulative statin dosages. We also resolved some limitations by employing alternative approaches. To minimize the influence of smoking, we adjusted for smoking-associated diseases (including COPD, ischemic heart disease, and stroke), indicating that the smoking status was relatively normalized in the 2 compared cohorts. To reduce the bias induced by cancer stages in the patients who used statins, we adjusted the cancer stages according to the treatment modalities. Our results show that the percentages of patients with lung cancer receiving dissimilar treatments were similar in the 2 cohorts (*P* = .4), signifying that the patients in both cohorts possibly had similar cancer stages. Although the number of patients who underwent surgery alone was slightly higher in the statin cohort (*n* = 726) than in the nonstatin cohort (*n* = 667), the statin cohort possibly included more patients with stage I cancer. However, patients treated by surgery alone constituted nearly 10% of the entire study population (*n* = 6270 for each cohort). Regarding the patients who underwent surgery, the difference in the HR values between the statin and nonstatin cohorts was not significant (Table 2). The potential bias associated with the concern that the patients visiting clinics and receiving treatment including statins were more likely to be diagnosed earlier and receive surgical interventions did not influence our observation. We also minimized the influence of alcohol-related diseases (including alcoholic psychoses, alcohol dependence syndrome, alcohol abuse, alcoholic fatty liver, acute alcoholic hepatitis, alcoholic cirrhosis, and alcoholic liver damage) in our analysis. However, other confounders (diet, physical activity, lipid profile, and body mass index) posed limitations in this study. Another limitation was the lack of information of cell types of lung cancer. We could not analyze the effects of statin use in different histology types. The potential analysis bias caused by these limitations was prevented by carefully enrolling the cohorts and controlling confounding factors such as comorbidities and treatment. The evidence-base and statistical method from a retrospective study using the insurance claims were lower than those from prospectively control study because the retrospective study might have some biases from the lack of the necessary adjustments for unmeasurable confounding factors. Moreover, the NHIRD did not allow us to identify individual patient, so the study could not to make sure if the patient received the drugs with good compliance by directly contacting the patient.

Overall, this population-based cohort study provides novel evidence that the all-cause mortality of lung cancer is substantially reduced in patients with a high cDDD of statins, regardless of the type of statin; statins potentially reduce lung cancer mortality. Additional prospective randomized controlled studies are required for verifying whether using statins reduces lung cancer mortality.

## MATERIALS AND METHODS

### Data source

Taiwan launched the single-payer compulsory National Health Insurance (NHI) program at the beginning of 1995 and has collected claims records covering all outpatient and inpatient medical benefit claims for nearly the entire population. The National Health Insurance Research Database (NHIRD) compiles the medical research and health claims data generated by the NHI program.

The NHIRD also includes the Registry of Catastrophic Illness Patient Database (RCIPD) for protecting vulnerable beneficiaries (including lung cancer patients) by exempting them from copayments for corresponding medical services. We used 2 data files: the registry of beneficiaries and the RCIPD. These data files were linked using an encrypted unique personal identification number to obtain the longitudinal medical history of each patient. This study was approved to fulfill the condition for exemption by the Institutional Review Board (IRB) of China Medical University (CMUH104-REC2-115). The IRB also specifically waived the consent requirement. A diagnostic code in the format of the International Classification of Diseases, Ninth Revision, Clinical Modification (ICD-9-CM) was used.

### Study population

**Figure 1:**
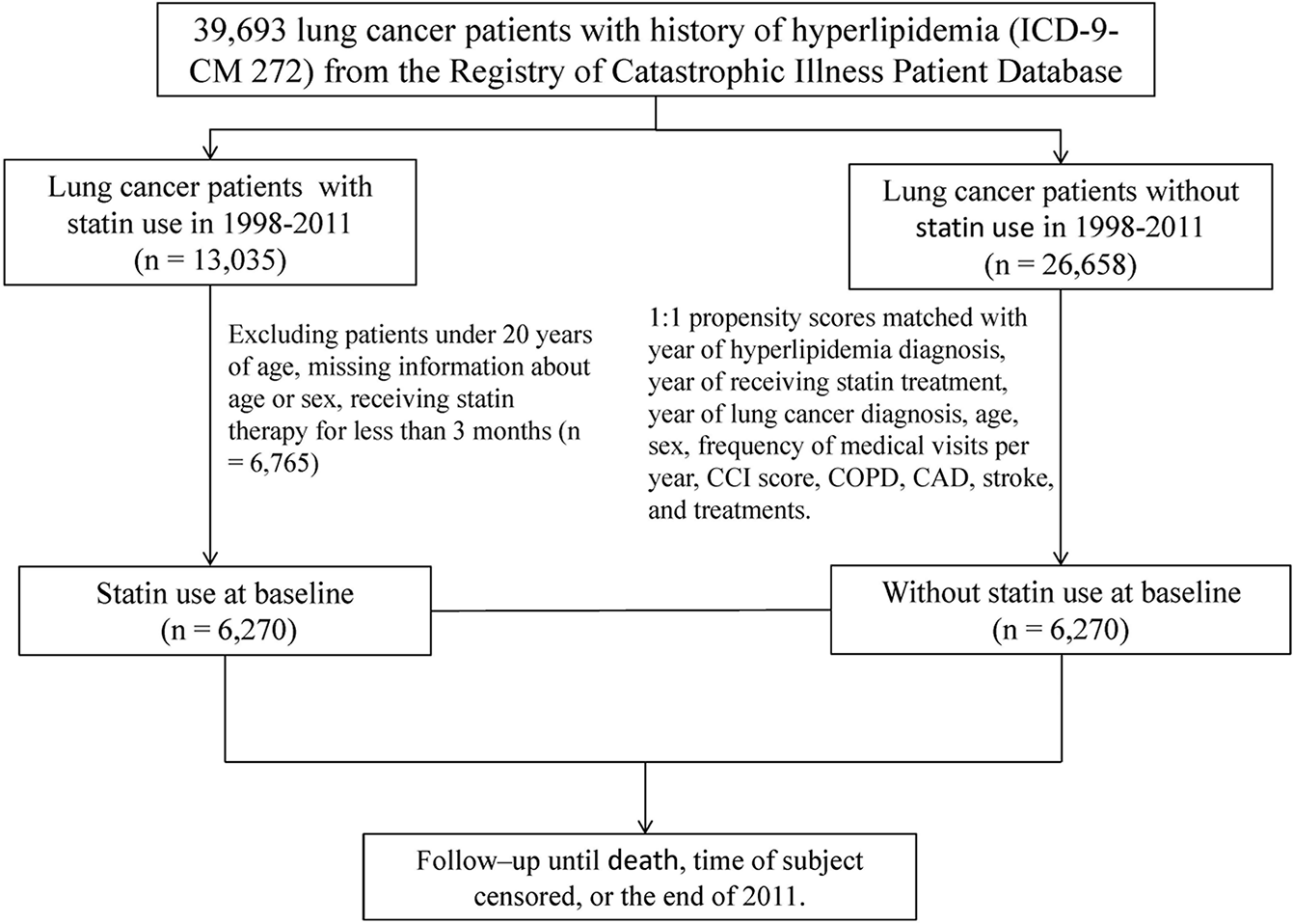
The flow chart demonstrating study design and cohorts' selection

From the RCIPD, we identified patients with hyperlipidemia (ICD-9-CM code 272) and newly diagnosed lung cancer (ICD-9-CM code 162) between January 1, 1998, and December 31, 2011. The diagnoses were recorded by related specialists and physicians according to the accurate pathological findings of qualified pathologists. Patients younger than 20 years and those with incomplete age or sex information were excluded. The patients were divided into 2 cohorts according to their statin use after hyperlipidemia diagnosis: a statin cohort comprising patients with lung cancer who received statin therapy for at least 3 months, and a nonstatin cohort composed of patients with lung cancer who received no statin therapy. We used the date on which statin treatment commenced as the index date. The patients with lung cancer receiving statin treatment and those without statin treatment were matched at a 1:1 ratio according to propensity scores. Figure 1 showed the flow chart demonstrating study design and cohorts' selection. The propensity scores were calculated through a logistic regression analysis to estimate the probability of the treatment assignment according to baseline variables, including the year of hyperlipidemia diagnosis; year of receiving statin treatment; year of lung cancer diagnosis; age; sex; frequency of medical visits per year (5 y before lung cancer diagnosis); Charlson comorbidity index (CCI) score; comorbidities of chronic obstructive pulmonary disease (COPD) (ICD-9-CM 491, 492, and 496), coronary artery disease (CAD) (ICD-9-CM 410–414), and stroke (ICD-9-CM 430–438); and treatments (palliative care, surgery, adjuvant therapy, radiotherapy, chemotherapy, and systemic therapy). The primary outcome was defined as the overall survival rate. All the patients were followed from the index date until death, withdrawal from the insurance system, or the end of 2011.

### Statistical analysis

The statin and nonstatin cohorts were matched according to the propensity scores. In brief, we conducted the analysis through the propensity score matching method in the SAS program. To estimate the propensity scores, a logistic regression model was used in which the statin status was regressed on the baseline characteristics listed in Table 1. The Mann–Whitney *U* test and chi-square test were used for quantifying differences in means or prevalence between the 2 cohorts for continuous or categorical matching variables, respectively. We measured the overall incidence; incidence by age, sex, CCI score, and comorbidities; and treatment for both cohorts. Cox proportional hazards models stratifying the matched pairs were used for estimating the hazard ratio (HR) and 95% confidence intervals (CIs) of mortality associated with the statin cohort compared with the nonstatin cohort. The cumulative defined daily dose (cDDD) was calculated by deriving the total prescribed DDD of each type of statin, namely simvastatin (ATC C10AA01), lovastatin (ATC C10AA02), pravastatin (ATC C10AA03), fluvastatin (ATC C10AA04), atorvastatin (ATC C10AA05), and rosuvastatin (ATC C10AA07), for statin users. For each statin type, the cDDD was partitioned into 2 levels by setting the cutoff value in the third quartile (highest 25%). Further analysis was conducted for assessing the association between the cDDD of each statin type and mortality. We divided the patients in the statin cohort into 4 groups by comparing their cDDD levels with the median cDDD value before and after lung cancer diagnosis. The patients with a cDDD level of statin use lower than the median were categorized into the low cDDD group, whereas those with a cDDD level of statin use higher than this value were placed into the high cDDD group. The Kaplan–Meier method was used to plot curves of the event-free rate among the groups, and a log-rank test was used to examine the difference between the curves. The follow-up time for the survival curves was calculated after lung cancer diagnosis. All analyses were conducted using SAS statistical software (Version 9.2 for Windows; SAS Institute, Inc., Cary, NC, USA). All statistical tests were executed at a 2-tailed significance level of .05.
